# Response Profiles of BV2 Microglia to IFN-γ and LPS Co-Stimulation and Priming

**DOI:** 10.3390/biomedicines11102648

**Published:** 2023-09-27

**Authors:** Meng Liy Pan, Nur Nabilah Ahmad Puzi, Yin Yin Ooi, Rajesh Ramasamy, Sharmili Vidyadaran

**Affiliations:** 1 Neuroinflammation Group, Immunology Laboratory, Department of Pathology, Faculty of Medicine and Health Sciences, Universiti Putra Malaysia, Serdang 43400, Malaysia; lilypan97@gmail.com (M.L.P.); puzi.nabilah@gmail.com (N.N.A.P.); 2 Department of Craniofacial Diagnostics & Biosciences, Faculty of Dentistry, Universiti Kebangsaan Malaysia, Jalan Raja Muda Abdul Aziz, Kuala Lumpur 50300, Malaysia; 3 School of Biosciences, Faculty of Health and Medical Sciences, Taylor’s University Lakeside Campus, 1, Jalan Taylor’s, Subang Jaya 47500, Malaysia; yinyin.ooi@taylors.edu.my; 4 Medical Advancement for Better Quality of Life Impact Lab, Taylor’s University Lakeside Campus, 1, Jalan Taylor’s, Subang Jaya 47500, Malaysia; 5 Stem Cell and Immunity Research Group, Immunology Laboratory, Department of Pathology, Faculty of Medicine and Health Sciences, Universiti Putra Malaysia, Serdang 43400, Malaysia; rajesh@upm.edu.my

**Keywords:** microglia, interferon gamma, lipopolysaccharide, reactive oxygen species, nitric oxide, neuroinflammation

## Abstract

(1) Background: The latest research illustrates that microglia phenotype is not the binary ‘resting’ and ‘activated’ profiles. Instead, there is wide diversity in microglia states. Similarly, when testing different stimulation protocols for BV2 microglia, we discovered differences in the response of the cells in terms of the production of intracellular ROS (iROS), nitric oxide (NO), CD40 expression, and migratory capacity. (2) Methods: BV2 microglia were treated with single interferon gamma (IFN-γ) stimulation, LPS/IFN-γ co-stimulation, and priming with IFN-γ followed by stimulation with LPS for 24 h. The responses of BV2 microglia were then assessed using the H_2_DCFDA test for iROS, the Griess assay for NO, immunophenotyping for CD40/CD11b/MHC II, and migration using a transwell apparatus. (3) Results: Single stimulation with IFN-γ induced NO but not ROS in BV2 microglia. Co-stimulation with LPS_200_IFN-γ_2.5_ induced a higher iROS production (a 9.2-fold increase) and CD40 expression (28031 ± 8810.2 MFI), compared to priming with _primed_IFN-γ_50_LPS_100_ (a 4.0-fold increase in ROS and 16764 ± 1210.8 MFI of CD40). Co-stimulation also induced cell migration. On the other hand, priming BV2 microglia (_primed_IFN-γ_50_LPS_100_) resulted in a higher NO production (64 ± 1.4 µM) compared to LPS_200_IFN-γ_2.5_ co-stimulation (44 ± 1.7 µM). Unexpectedly, priming inhibited BV2 migration. (4) Conclusions: Taken together, the findings from this project reveal the ability of co-stimulation and priming in stimulating microglia into an inflammatory phenotype, and the heterogeneity of microglia responses towards different stimulating approaches.

## 1. Introduction

BV2 microglia are a v-raf/v-myc-immortalised murine cell line [1] commonly used in in vitro studies of microglia. They can be stimulated into a reactive, inflammatory phenotype using various stimulators, including lipopolysaccharide (LPS) and interferon gamma (IFN-γ).

Microglia develop from yolk sac hematopoietic progenitors [2,3] and have important homeostatic and immune functions within the central nervous system (CNS). In brain development, microglia regulate the size of the neural precursor pool by inducing neural precursor death [4] and phagocytosis [5], pruning synapses [6,7], and govern the wiring of the embryonic [8] and postnatal [9] brain. Infection or tissue injury in the brain triggers microglia into a ‘reactive’ phenotype, with microglia expressing reactive oxygen species (ROS), nitric oxide (NO), and proinflammatory cytokines. Recently studies have revealed that microglia in the mouse [10] and human [11] brain are in a much diverse cell state than previously thought. Instead of the dichotomous ‘resting’ and ‘activated’ phenotypes of microglia, the studies reveal up to nine transcriptionally distinct microglial subtypes that shift with age and injury states. Differences in microglia reactivity to in vitro stimulation are also apparent. For instance, ATP induces BV2 microglia migration [12], but not NO (unpublished data). Noting the differences in stimulation profiles will assist with selecting the ideal stimulant for the purposes of a particular study and, more importantly, to determine the receptor-induced cell signaling that leads to each reactive phenotype.

Here, we test different microglia stimulation protocols to stimulate BV2 cells into an inflammatory and potentially neurotoxic phenotype. Our laboratory is developing a culture model of microglia-induced neuronal damage, and we seek to profile the responses of BV2 microglia to IFN-γ alone, LPS /IFN-γ co-stimulation, and IFN-γ priming followed by LPS stimulation. LPS is a component of a Gram-negative bacteria cell wall that activates microglia into an inflammatory phenotype via toll-like receptor 4 (TLR4)-signaling. IFN-γ is a cytokine expressed as a consequence of tissue damage. It represents an endogenous response to tissue damage and activates microglia and other tissue macrophages. Both LPS and IFN-γ have been used individually [13,14,15,16] and in combination [17,18] to stimulate microglia in vitro and in vivo. The response profiles of BV2 cells to the different stimulation protocols were determined by their iROS and NO production, expression of surface markers CD40/CD11b/MHC II, as well as the ability to migrate towards the stimulants. Experiments were performed concurrently with 1000 ng/mL LPS and single doses of IFN-γ and LPS at the respective concentrations for comparison. We intend to develop a culture model of microglia-mediated neurotoxicity, and the iROS and NO parameters may be indicative of subsequent neurotoxic effects of microglial inflammatory responses.

## 2. Materials and Methods

### 2.1. BV2 Microglia Cell Culture

The BV2 microglia cell line was a generous gift from Prof. Dr. Johnson Stanslas, Universiti Putra Malaysia. The cells were cultured in Dulbecco modified Eagle medium with 5% heat-inactivated foetal bovine serum, 100 U/mL penicillin, and 100 μg/mL streptomycin, 1.25 μg/mL amphotericin B, 0.01 μg/mL gentamycin, 1× non-essential amino acid (all from Gibco; Thermo Fisher Scientific, Inc., Waltham, MA, USA), 6.25 μg/mL insulin, and 1.5 g/L sodium bicarbonate (both from Sigma-Aldrich, St. Louis, MO, USA). Cultures were maintained at 37 °C with 95% humidified air and 5% CO_2_. Cells were trypsinised with 0.25% trypsin-EDTA (Gibco; Thermo Fisher Scientific, Inc., Waltham, MA, USA) for 5 min at 37 °C. Cell viability was determined using the trypan blue exclusion assay.

### 2.2. BV2 Microglia Stimulation Protocols

#### 2.2.1. Single Stimulation with IFN-γ

BV2 microglia cells were seeded at 6.25 × 10^4^ cells/cm^2^ in 96-well plates and incubated at 37 °C with 95% humidified air and 5% CO_2_ overnight for cell attachment. The next day, supernatant was removed and added with media containing 2.5, 5, or 10 ng/mL of recombinant IFN-γ (R&D system Inc., Minneapolis, MN, USA; Cat. No.: 485-MI) and incubated for 24, 48, and 72 h.

#### 2.2.2. LPS/IFN-γ Co-Stimulation

**Figure 1:**
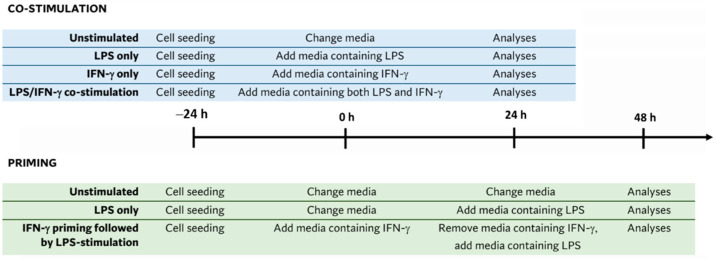
Schematic representation of co-stimulation and priming protocols for BV2 cultures. Diagram outlines the timing of the cell culture and stimulation, duration, and time of analyses.

Figure 1 is a schematic representation of the co-stimulation and priming protocol for the BV2 cultures.

Co-stimulation was performed by simultaneously adding LPS (Sigma-Aldrich, St. Louis, MO, USA; Cat. No.: L8274) and recombinant IFN-γ to BV2 cells. Three different co-stimulation concentrations were used, namely 10 ng/mL LPS and 10 ng/mL IFN-γ (LPS_10_IFN-γ_10_), 100 ng/mL LPS and 5 ng/mL IFN-γ (LPS_100_IFN-γ_5_), and 200 ng/mL LPS and 2.5 ng/mL IFN-γ (LPS_200_IFN-γ_2.5_). Single stimulation with 10, 100, and 200 ng/mL LPS or 2.5, 5, and 10 ng/mL IFN-γ were also tested to determine the augmentation of ROS or NO production after adding both stimulants. LPS 1000 ng/mL was used as a positive control.

BV2 cells were seeded at 6.25 × 10^4^/cm^2^ in a 96-well plate and incubated at 37 °C with 95% humidified air and 5% CO_2_ overnight. The supernatant was removed, and media containing LPS (10, 100, 200, 1000 ng/mL), IFN-γ (2.5, 5, 10 ng/mL), or LPS/IFN-γ (LPS_10_IFN-γ_10_, LPS_100_IFN-γ_5_, and LPS_200_IFN-γ_2.5_) were added for 24 h.

#### 2.2.3. IFN-γ Priming Followed by Stimulation with LPS

Priming was performed by adding DMEM containing IFN-γ into BV2 cells for 24 h, followed by changing the supernatant with media containing LPS for another 24 h. Cells stimulated with LPS alone were included as the control.

BV2 cells were seeded at 3.125 × 10^4^ cells/cm^2^ in a 96-well plate and incubated at 37 °C with 95% humidified air and 5% CO_2_ overnight. For unstimulated cells, a media change was performed twice, at the first and second 24 h. For cells stimulated with LPS alone, spent media were changed with fresh media for the first 24 h, followed by media containing respective doses of LPS for the second 24 h. For priming, cells were added with media containing 10 ng/mL IFN-γ (_primed_IFN-γ_10_) or 50 ng/mL IFN-γ (_primed_IFN-γ_50_) for the first 24 h. The supernatant was then removed, and media containing 10 ng/mL LPS (LPS_10_), 100 ng/mL LPS (LPS_100_), 200 ng/mL LPS (LPS_200_), or 1000 ng/mL LPS (LPS_1000_) were added for the subsequent 24 h.

### 2.3. Intracellular ROS Measurement

Intracellular ROS was detected using 2′,7′-dichlorodihydrofluorescein diacetate assay (H_2_DCFDA; Invitrogen; Thermo Fisher Scientific, Inc., Waltham, MA, USA; Cat. No.: D399). Briefly, stimulated BV2 cells were washed twice with 1× PBS. Cells were incubated with 20 μM of H_2_DCFDA reagent in 100 μL of phenol red-free DMEM for 1 h, at 37 °C with 95% humidified air and 5% CO_2_. Then, the supernatant was discarded, and cells were washed twice with 1× PBS, followed by an addition of 100 μL phenol red-free DMEM. Fluorescence was quantitated using a Hybrid multi-mode microplate reader (Synergy H1, BioTek, Winooski, VT, USA), at ex/em of 493 nm/520 nm. Results are reported in Relative Fluorescence Units (RFUs). RFUs’ values of all wells were deducted with RFUs’ values of phenol red-free DMEM, which serves as the background reading.

### 2.4. NO Measurement

NO was detected in the supernatant of BV2 cells using the Griess assay. Fifty microlitres of the supernatant from BV2 cells were transferred into a 96-well plate in triplicate, and the same volume of the Griess reagent was added (both 1% sulphanilamide and 0.1% NED dissolved in 2.5% phosphoric acid) (all from Sigma-Aldrich, St. Louis, MO, USA). The plate was incubated at room temperature for 10 min, and absorbance was read at 530 nm with a microplate reader. Nitrite concentration was calculated using the formula generated from a standard curve of two-fold serial diluted sodium nitrite (NaNO_2_; 100, 50, 25, 12.5, 6.25, 3.125, 1.5625, and 0 μM). Absorbance values of all wells were deducted with the absorbance of 0 μM of NaNO_2_ to eliminate the background reading. The results are displayed as concentration of NO_2_^−^ in μM.

### 2.5. Immunophenotyping

BV2 cells were immunophenotyped against three surface markers, which were CD40 (Cat. No.: 558695), CD11b (Cat. No.: 553310), and MHC II (Cat. No.: 554929) (all from BD Biosciences, Franklin Lakes, NJ, USA). BV2 cells were seeded at 6.25 × 10^4^ cells/cm^2^ and 3.125 × 10^4^ cells/cm^2^ in a 6-well plate for co-stimulation and priming, respectively, followed by incubating the cells at 37 °C with 95% humidified air and 5% CO_2_ overnight. Cells were stimulated with LPS_10_IFN-γ_10_, LPS_200_IFN-γ_2.5_, _primed_IFN-γ_10_LPS_100_, or _primed_IFN-γ_50_LPS_100_ as described in Section 2.2.2 and Section 2.2.3. Following stimulation, cells were trypsinised using 0.25% trypsin-EDTA for 5 min at 37 °C, and the pellet was resuspended in 0.2% BSA in 1× PBS. Cell viability was determined using trypan blue exclusion assay.

Cells were distributed at a density of 5.0 × 10^5^ cells/tube and washed with 1 mL of 0.2% BSA in 1× PBS twice, followed by incubating with 5 μL of each antibody in 100 μL of 0.2% BSA in 1× PBS, at 4 °C for 30 min. Cells were again washed twice and resuspended with 500 μL of 0.2% BSA in 1× PBS. Ten thousand gated events were recorded. For each antibody, gating was determined based on appropriate isotype-stained controls. An unstained sample was prepared to reveal cellular autofluorescence to exclude it as the background. Data were analysed using BD FACSDiva™ Software version 8.0.

### 2.6. Transwell Migration Assay

The transwell migration assay was carried out with polycarbonate cell culture inserts with a pore size of 8 μm (Falcon; Corning Inc., Corning, NY, USA; Cat. No.: 353097) in a 24-well plate. For LPS/IFN-γ co-stimulation, BV2 cells were seeded (3.33 × 10^5^/cm^2^) in transwell inserts in 500 µL of serum-free media. The 24-well plate was incubated at 37 °C with 5% carbon dioxide for 30 min for cell adherence. Then, 500 µL of a stimulant containing LPS_10_IFN-γ_10_ or LPS_200_IFN-γ_2.5_ in serum-free media was added to the 24-well plate.

For priming, BV2 cells were seeded (3.125 × 10^4^/cm^2^) in a 6-well plate and incubated at 37 °C with 95% humidified air and 5% CO_2_ overnight. The supernatant was then removed, and cells were primed with media containing 10 or 50 ng/mL of IFN-γ for 24 h. IFN-γ primed cells were trypsinised and seeded at 3.33 × 10^5^/cm^2^ in the transwell inserts in 500 µL of serum-free media. The 24-well plate was incubated at 37 °C with 5% carbon dioxide for 30 min for cell adherence. A stimulant containing 100 ng/mL LPS in serum-free media was added to the 24-well plate.

Cells were incubated in the incubator for 12 h to allow the cells to migrate across the polycarbonate cell culture insert. Then, the supernatant in the transwell insert was discarded, and the transwell inserts were rinsed twice with 1× PBS. The transwell inserts were fixed using 2% paraformaldehyde (PFA) for 1 h, and cells were permeabilised using 0.01% Triton-X for another 1 h, followed by staining the cells using crystal violet (Sigma-Aldrich, St. Louis, MO, USA; Cat. No.: 111885) for 30 min. The transwell inserts were rinsed twice in 1× PBS between the fixation, permeabilization, and staining steps. Once the transwell inserts were free from excess stain, unmigrated cells were swabbed carefully using a cotton bud dipped into 1× PBS. The membranes of the transwell inserts were cut with a scalpel and mounted on a microscope slide with DPX. Migrated cells were then viewed under the microscope and representative images were taken. Semi-quantitative scoring was performed blinded by two investigators to estimate the extent of migration.

### 2.7. Statistical Analysis

Statistical analysis was carried out in GraphPad Prism 8.0.1 (San Diego, CA, USA). Significance was assessed using one-way analysis of variance (ANOVA) followed by the Tukey’s post hoc test.

## 3. Results

### 3.1. LPS/IFN-γ Co-Stimulation

A single dose of IFN-γ stimulated NO production by BV2 microglia (Figure S1) but did not increase perceivable amounts of iROS (Figure S2). LPS (1000 ng/mL) induced 6606 ± 244.1 RFU iROS at 24 h, which then decreased at 48 h to 2689 ± 45.2 RFU and 1503 ± 130.5 RFU at 72 h (Figure S2). The 24 h timepoint was thereafter chosen for future iROS analysis.

**Figure 2:**
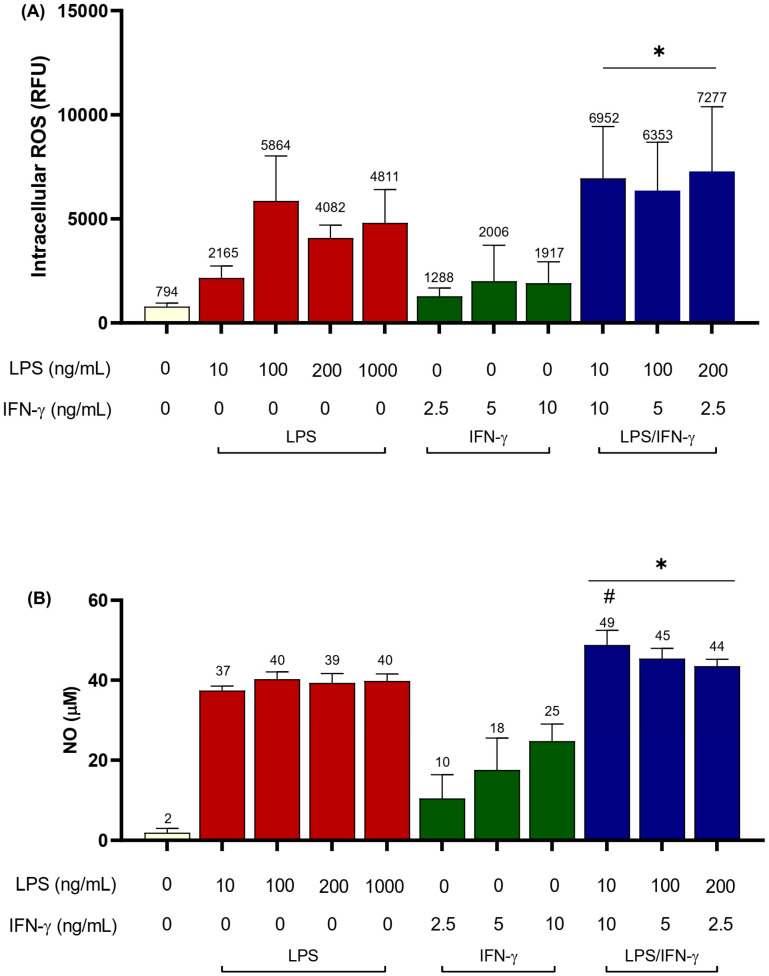
Intracellular ROS and NO production following LPS/IFN-γ co-stimulation in BV2 microglia. BV2 cells (6.25 × 10^4^ cells/cm^2^) were seeded in a 96-well plate and incubated with LPS (10, 100, 200, 1000 ng/mL), IFN-γ (2.5, 5, 10 ng/mL), or LPS/IFN-γ (LPS_10_IFN-γ_10_, LPS_100_IFN-γ_5_, and LPS_200_IFN-γ_2.5_), for 24 h. (**A**) iROS was determined using the H_2_DCFDA assay, and (**B**) NO was determined using the Griess assay. Results are expressed as mean ± SD of three independent experiments with at least three replicates. * *p* < 0.0001 compared to unstimulated cells; # *p* < 0.001 compared to 1000 ng/mL LPS-stimulated cells; one-way ANOVA with Tukey’s post hoc test.

LPS/IFN-γ co-stimulation induced high amounts of iROS in BV2 cells compared to unstimulated cells (Figure 2A; *p* < 0.0001). The highest iROS levels corresponded with LPS_200_IFN-γ_2.5_ with 7277 ± 3104.3 RFUs, a 9.2-fold increase in iROS compared to unstimulated cells. All LPS/IFN-γ co-stimulation concentrations induced greater iROS production compared to single stimulation with corresponding doses of LPS or IFN-γ, although not all were statistically different (Figure 2A). For instance, cells stimulated with LPS_200_ induced iROS levels of 4082 ± 615.1 RFUs and IFN-γ_2.5_ alone only induced 1288 ± 388.9 RFUs (*p* < 0.01).

LPS/IFN-γ co-stimulation also induced NO production in BV2 cells (Figure 2B; *p* < 0.0001). LPS_10_IFN-γ_10_ induced the highest NO of 49 ± 3.7 µM, higher compared to 1000 ng/mL LPS (40 ± 1.8 µM; *p* < 0.001).

**Figure 3:**
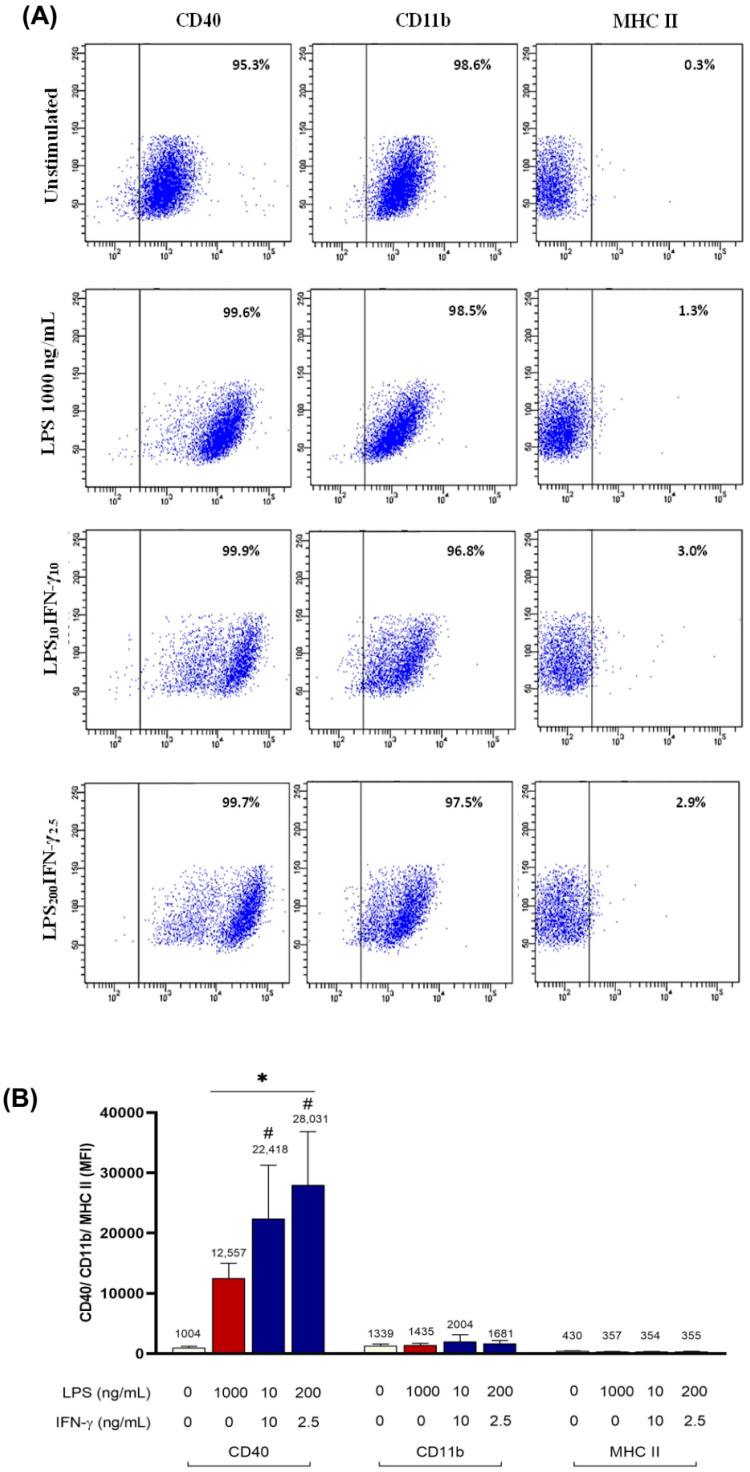
CD40, CD11b, and MHC II expression following LPS/IFN-γ co-stimulation of BV2 microglia. BV2 cells (6.25 × 10^4^ cells/cm^2^) were seeded in a 6-well plate and stimulated with 1000 ng/mL LPS, LPS_10_IFN-γ_10_, and LPS_200_IFN-γ_2.5_ for 24 h. Cells positive for CD40, CD11b, and MHC II were determined by flow cytometry. (**A**) Numbers in the right quadrant within each dot plot indicate the percentage of BV2 cells positive for each marker. Dot plots are representative of three independent experiments. (**B**) Median fluorescence intensity (MFI) of CD40, CD11b, and MHC II of BV2 microglia. Results are expressed as mean ± SD of three independent experiments. * *p* < 0.0001 compared to unstimulated cells. # *p* < 0.0001 compared to 1000 ng/mL LPS-stimulated cells; one-way ANOVA with Tukey’s post hoc test.

The co-stimulation protocols that induced the highest iROS and NO were then further characterised by examining phenotypic markers of microglia. Triple-staining immunophenotyping with CD40, CD11b, and MHC II markers was performed. Gating strategy and isotype controls used for immunophenotyping are demonstrated and described in (Figure S3). All cells regardless of stimulation had more than95% of the cell population positive for CD40 and CD11b. However, less than 5% of cells expressed MHC II in all groups (Figure 3A). LPS_200_IFN-γ_2.5_ induced the highest expression of CD40 (28031 ± 8810.2 MFI), compared to 1000 ng/mL LPS (12557 ± 2440.8 MFI) (Figure 3B; *p* < 0.0001). However, changes in MFI of CD11b and MHC II remained unremarkable.

**Figure 4:**
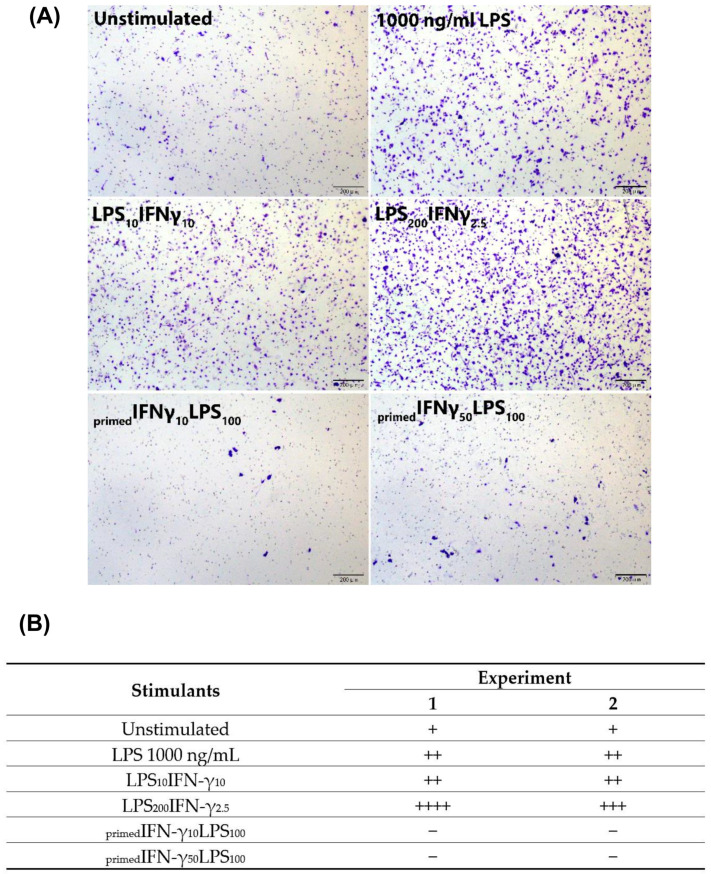
**BV2 microglia migration following LPS/IFN-γ co-stimulation and _primed_IFN-γ/LPS stimulation.** For co-stimulation, BV2 cells (3.33 × 10^5^/cm^2^) were seeded in transwell inserts and placed in a 24-well plate containing LPS_10_IFN-γ_10_ or LPS_200_IFN-γ_2.5_. For priming, BV2 cells (3.125 × 10^4^/cm^2^) were seeded in a 6-well plate and incubated overnight. BV2 cells were then primed with IFN-γ_10_ or IFN-γ_50_ for 24 h. IFN-γ primed cells were trypsinised and seeded at 3.33 × 10^5^/cm^2^ in transwell inserts and placed in a 24-well plate containing 100 ng/mL LPS. Migration was assessed at 12 h. (**A**) Representative fields of migrated cells for each stimulation protocol. (**B**) Semi-quantitative scores of BV2 migration from two independent experiments. Semi-quantitative scoring of migration indicated with + (low number of migrated cells), ++ (moderate number of migrated cells), +++ (high number migrated cells), ++++ (very high number of migrated cells), − (negligible number of migrated cells).

The two co-stimulation doses also caused BV2 cell migration, with LPS_200_IFN-γ_2.5_ inducing the most migration (Figure 4).

### 3.2. IFN-γ Priming Followed by LPS Stimulation

**Figure 5:**
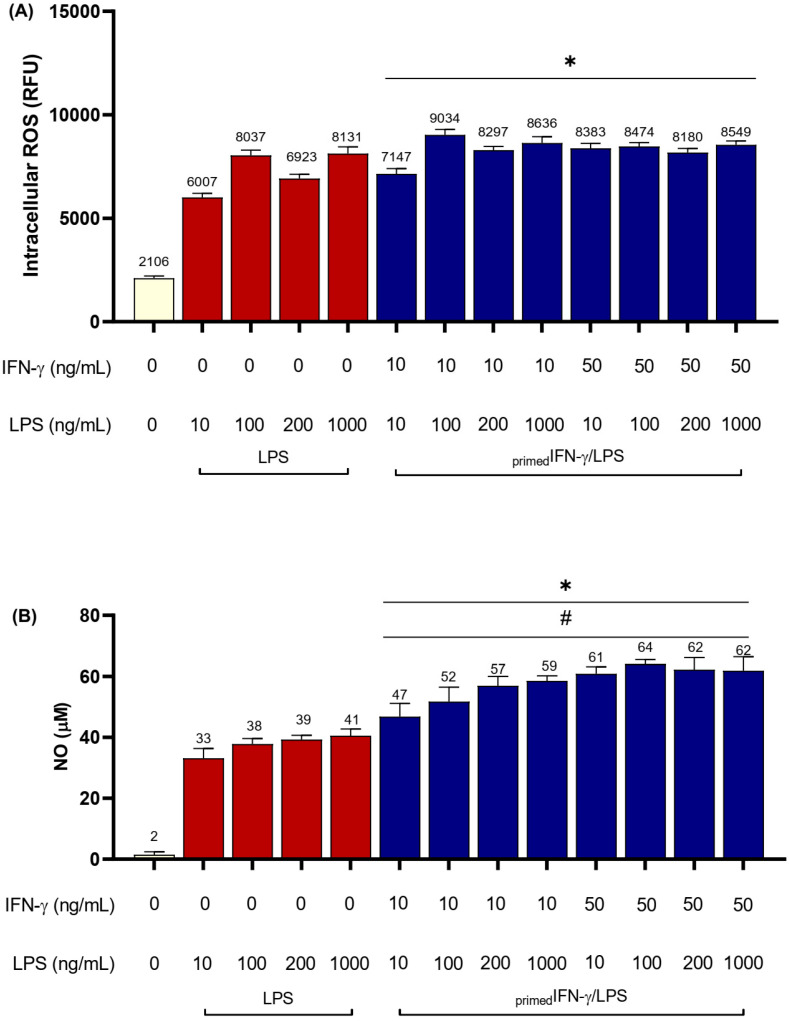
Intracellular ROS and NO production of _primed_IFN-γ/LPS in BV2 microglia. BV2 cells (3.125 × 10^4^ cells/cm^2^) were seeded in a 96-well plate and primed with IFN-γ_10_ or IFN-γ_50_ for 24 h. Supernatant was removed, and cells were subsequently stimulated with LPS_10_, LPS_100_, LPS_200_, and LPS_1000_ for another 24 h. Cells were also stimulated with corresponding doses of LPS without IFN-γ priming. (**A**) iROS was determined using the H_2_DCFDA assay, and (**B**) NO was determined using the Griess assay. Results are expressed as mean ± SD of three independent experiments with at least three replicates. * *p* < 0.0001 compared to unstimulated cells. # *p* < 0.001 compared to 1000 ng/mL LPS-stimulated cells; one-way ANOVA with Tukey’s post hoc test.

All primed protocols induced iROS in BV2 cells (Figure 5A; *p* < 0.0001), with a ~4-fold increase compared to unstimulated cells. _primed_IFN-γ_10_LPS_100_ induced the highest iROS levels of 9034 ± 882.6 RFUs. Interestingly, priming BV2 microglia with IFN-γ prior to LPS stimulation did not induce a remarkable iROS increase compared to cells without IFN-γ priming.

Priming microglia with IFN-γ did, however, induce higher NO compared to cells without IFN-γ priming (*p* < 0.0001). The highest increment was observed in _primed_IFN-γ_50_LPS_10_ as compared to LPS_10_, where NO increased by 84.4% (from 33 ± 3.2 µM to 61 ± 2.28 µM). Also, the highest NO concentration was induced with _primed_IFN-γ_50_LPS_100,_ with NO levels of 64 ± 1.4 µM, compared to unstimulated cells (2 ± 0.9 µM) (Figure 5B).

**Figure 6:**
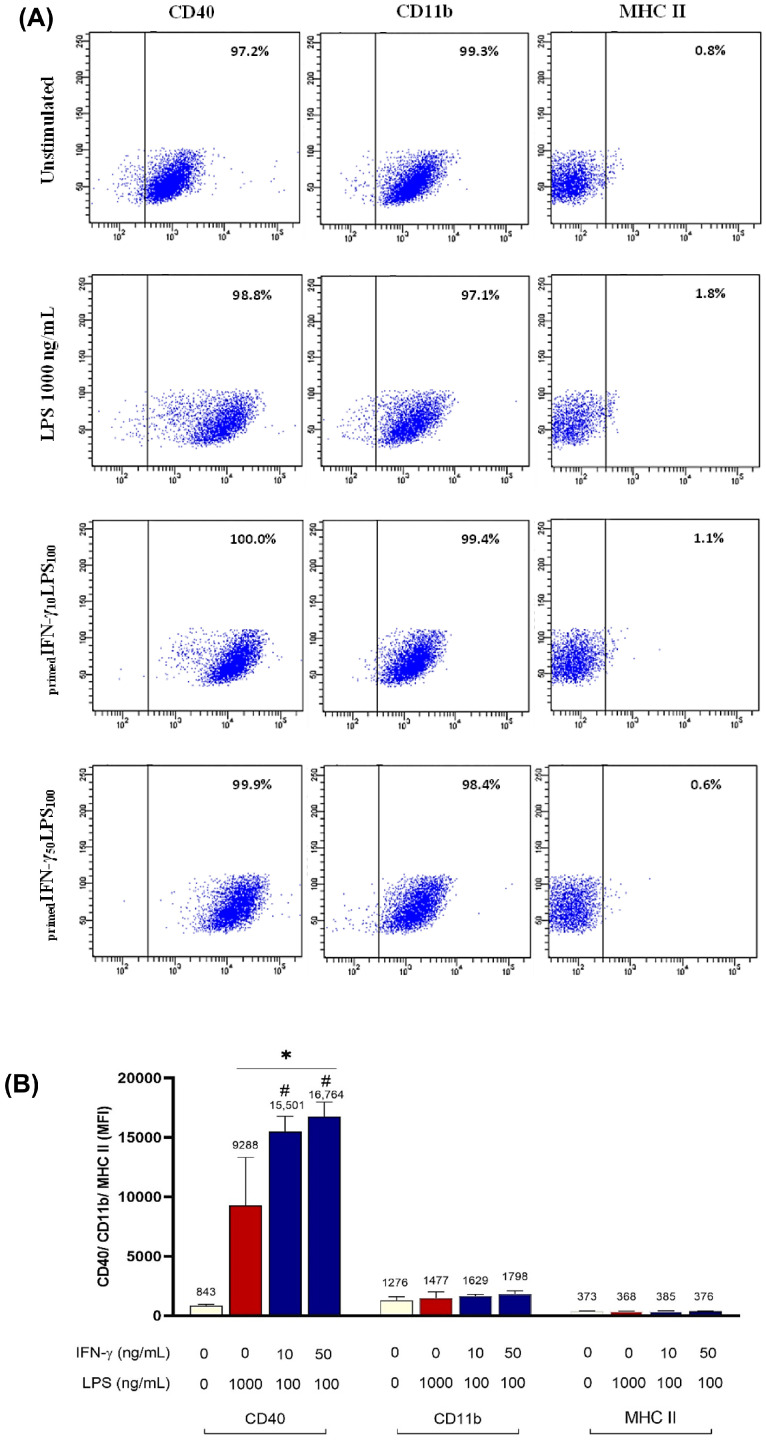
CD40, CD11b, and MHC II expression of _primed_IFN-γ/LPS of BV2 microglia. BV2 cells (3.125 × 10^4^ cells/cm^2^) were seeded in a 6-well plate and primed with IFN-γ_10_ or IFN-γ_50_ for 24 h. The supernatant was removed, and cells were subsequently stimulated with LPS_100_ for another 24 h. Cells positive for CD40, CD11b, and MHC II were determined by flow cytometry. (**A**) Numbers in the right quadrant within each dot plot indicate the percentage of BV2 cells positive for each marker. Dot plots are representative of three independent experiments. (**B**) Median fluorescence intensity (MFI) of CD40, CD11b, and MHC II of BV2 microglia. Results are expressed as mean ± SD of three independent experiments. * *p* < 0.0001 compared to unstimulated cells. # *p* < 0.0001 compared to 1000 ng/mL LPS-stimulated cells; one-way ANOVA with Tukey’s post hoc test.

These priming concentrations were immunophenotyped against CD40/CD11b/MHC II markers. The gating strategy used for immunophenotyping is demonstrated in (Figure S3). More than 90% of _primed_IFN-γ/LPS cells expressed CD40 and CD11b, similar to unstimulated and LPS-stimulated cells (Figure 6A). However, less than 5% of _primed_IFN-γ/LPS cells expressed MHC II. All _primed_IFN-γ/LPS were able to induce CD40 expression in BV2 cells (Figure 6A; *p* < 0.0001). _primed_IFN-γ_50_LPS_100_ induced the highest CD40 expression with 16764 ± 1210.8 MFI, compared to 1000 ng/mL LPS with 9288 ± 4024.2 MFI (Figure 6B; *p* < 0.0001). Meanwhile, changes in MFI of CD11b and MHC II remained unremarkable. Interestingly, priming BV2 cells appeared to inhibit their migration (Figure 4).

To summarise, co-stimulation induced higher iROS and CD40 expression than priming. Of the two co-stimulation concentrations, LPS_200_IFN-γ_2.5_ induced the highest iROS increase of 9.2-fold (*p* < 0.001). Meanwhile, _primed_IFN-γ_50_LPS_100_ induced the highest NO levels in BV2 cells. However, _primed_IFN-γ inhibits migration of BV2 cells towards LPS (Table 1).

## 4. Discussion

Three different protocols for stimulating BV2 microglia using IFN-γ and LPS, namely single IFN-γ stimulation, LPS/IFN-γ co-stimulation, and priming with IFN-γ followed by stimulation with LPS, were investigated. A single dose of IFN-γ (2.5, 5 or 10 ng/mL) induced NO production in our BV2 cultures but failed to induce intracellular ROS (iROS). Spencer and colleagues also reported a lack of iROS production with 10 and 50 ng/mL IFN-γ [19]. It appears that IFN-γ receptor signaling alone appears unremarkable for iROS production in BV2 microglia, although it induces NO production.

Although LPS can induce iROS expression, the addition of IFN-γ (in the co-stimulation protocol) boosts iROS levels. Adding 10 ng/mL IFN-γ with 10 ng/mL LPS (LPS_10_IFN-γ_10_) boosted BV2 microglia production of iROS by 3.2-fold, whilst 2.5 ng/mL IFN-γ added with 200 ng/mL LPS increased ROS levels by 1.8-fold compared to LPS alone at the corresponding doses. Priming microglia with IFN-γ, however, did not augment LPS-induced iROS levels (a 1.1- to 1.4-fold change). This contradicts Spencer et al. [19], who primed BV2 microglia with IFN-γ, followed by stimulation with ATP. They demonstrated that 10 ng/mL and 50 ng/mL of IFN-γ increased iROS production in ATP-stimulated BV2 cells by 3.2-fold and 9.3-fold, respectively. However, it is noteworthy that ATP alone did not induce iROS [19]. It appears that LPS, but not ATP, is a good stimulator of iROS production.Priming BV2 microglia with IFN-γ led to significantly higher NO amounts in LPS-stimulated cells compared to cells stimulated with LPS alone. The priming effect of IFN-γ on NO has been described [20,21]. Pre-treatment of murine macrophage RAW 264.7 cells with IFN-γ augmented LPS-induced NF-κB activation and was accompanied by increased nitrite production. As described above, the same augmentation does not seem to occur for iROS expression indicating that the IFN-γ priming effect may not occur for all LPS-induced responses [22] and that the priming effect varies depending on the secondary stimuli. Interestingly for co-stimulation, the highest NO production was induced by LPS_10_IFN-γ_10_, whilst the highest iROS was induced by LPS_200_ IFN-γ_2.5_, demonstrating that the concentration that yielded the highest iROS did not necessarily cause the highest NO production, and vice versa. Similarly, _primed_IFN-γ_10_LPS_100_ induced the highest ROS, and _primed_IFN-γ_50_LPS_100_ induced the highest NO.

Our aim is to stimulate microglia into an inflammatory, neurotoxic phenotype for developing a culture model of microglia-induced neuronal damage. Interestingly, Papageorgiou and colleagues demonstrated on hippocampal slice cultures that, although chronic activation with LPS renders the microglia reactive, neuronal damage is caused only in the presence of the IFN-γ receptor signaling [23]. Importantly, they go on to demonstrate that NO is responsible for neuron damage. LPS is a component of the cell wall of Gram-negative bacteria and represents an exogenous, infectious agent. It induces reactive phenotypes of microglia through TLR4 signaling and activation of the NFκB inflammatory pathway [24]. IFN-γ, on the other hand, is an inflammatory cytokine and part of the endogenous tissue response to inflammation. There are also aging-related increases in IFN-γ. IFN-γ binds to the IFN-γ receptor present on microglia to activate multiple effector genes, including inducible nitric oxide synthase (iNOS), and CD40 through the JAK/STAT pathway [25]. LPS and IFN-γ synergistically up-regulate proteins of the TLR4 signaling pathway, namely CD14, TLR4, MD-2, and MyD88 expression [24]. Therefore, when both IFN-γ and LPS signaling is induced, for instance, in our LPS/IFN-g co-stimulation protocol, it causes amplification of STAT1 activation. Thus, co-stimulation involves up-regulation of both NF-κB and STAT1.

In our study, _primed_IFN-γ_50_LPS_100_ induced the highest NO levels across all stimulation protocols tested. All other tested parameters, namely iROS, CD40 expression, and migration, were highest with LPS_200_IFN-γ_2.5_ co-stimulation_._ For priming, namely when IFN-g administered prior LPS stimulation, IFN-γ may act by upregulating CD14, increasing its sensitivity towards secondary stimulus, and augmenting NF-κB activation. Unexpectedly, priming BV2 microglia inhibited BV2 cells migration towards LPS. Interestingly, migration of BV2 cells is enhanced when IFN-γ is added simultaneously with LPS. IFN-γ, therefore, can have opposing effects on cell migration depending on the stimulation protocol. The literature demonstrates several different effects of priming with IFN-γ on migration. IFN-γ-primed macrophages have enhanced migration towards chemokine CCL2/MCP-1 [26], although it had negligible effect on migration towards adenosine diphosphate (ADP) [27]. Rat microglia primed with IFN-γ decreased migration of microglia in response to zymosan-activated serum [28]. Additionally, IFN-γ priming drastically suppressed CCL2-induced primary human monocyte migration. Notably, inhibition of migration increased with the IFN-γ pre-incubation duration [29].

## 5. Conclusions

The activation state of microglia varies depending on stimulation protocols. LPS/IFN-γ co-stimulation induced higher ROS production, CD40 expression, and migration compared to IFN-γ-priming of LPS-stimulated BV2 cells. However, priming resulted in a higher NO production compared to co-stimulation, and migration was inhibited in IFN-γ-primed BV2 microglia cells.

## Figures and Tables

**Table 1:** Summary of findings of IFN-γ single stimulation, LPS/IFN-γ co-stimulation, and _primed_IFN-γ /LPS priming protocols on iROS and NO production, CD40 expression and migration in BV2 cells. Difference between the highest induction value (grey column) was compared with the other co-stimulation and priming values and tested for significance.

	Unstimulated	LPS 1000 ng/mL	IFN-γ 2.5 ng/mL	IFN-γ 5 ng/mL	IFN-γ 10 ng/mL	LPS_10_IFN-γ_10_	LPS_200_IFN-γ_2.5_	_primed_IFN-γ_10_LPS_100_	_primed_IFN-γ_50_LPS_100_
**iROS (fold increase)**	1	3–6	1.1	0.7	1.4	8.8	9.2 *	4.3	4.0
**NO (µM)**	2	40–41	10 ± 5.9	18 ± 7.9	25 ± 4.2	49 ± 3.7	44 ± 1.7	52 ± 4.8	64 ± 1.4 **
**CD40 (MFI)**	800–1000	9000–13,000	N/A	N/A	N/A	22,418 ± 8846.6	28,031 ± 8810.2	15,501 ± 1274.9	16,764 ± 1210.8
**Migration**	+	++	N/A	N/A	N/A	++	+++	−	−

Columns shaded with grey indicate the highest induction for each of the measured parameters across all stimulation protocols. Semi-quantitative scoring of migration was indicated with + (low number of migrated cells), ++ (moderate number of migrated cells), +++ (high number migrated cells), − (negligible number of migrated cells). * *p* < 0.0001 compared to _primed_IFN-γ_10_LPS_100_, _primed_IFN-γ_50_LPS_100_. ** *p* < 0.0001 compared to LPS_10_IFN-γ_10_, LPS_200_IFN-γ_2.5_, _primed_IFN-γ_10_LPS_100_.

## Data Availability

The data presented in this study are available on request from the corresponding author.

## Supplementary Materials

The following supporting information can be downloaded at: https://www.mdpi.com/article/10.3390/biomedicines11102648/s1, Figure S1: Single-dose IFN-γ (2.5, 5, 25, 50, 100 ng/mL) stimulation and NO production in BV2 microglia; Figure S2: IFN-γ does not stimulate substantial amounts of intracellular ROS; Figure S3: Isotype control and gating strategy for immunophenotyping analysis of BV2 cells.
