# Supplementary material for: Response Profiles of BV2 Microglia to IFN-γ and LPS Co-Stimulation and Priming
Source: Biomedicines. 2023 Sep 27;11(10):2648. doi: 10.3390/biomedicines11102648 (PMC10604055; doi:10.3390/biomedicines11102648)
Supplement: Supplementary file 1 [file biomedicines-11-02648-s001.zip › Figure S1A & S1B.pdf]

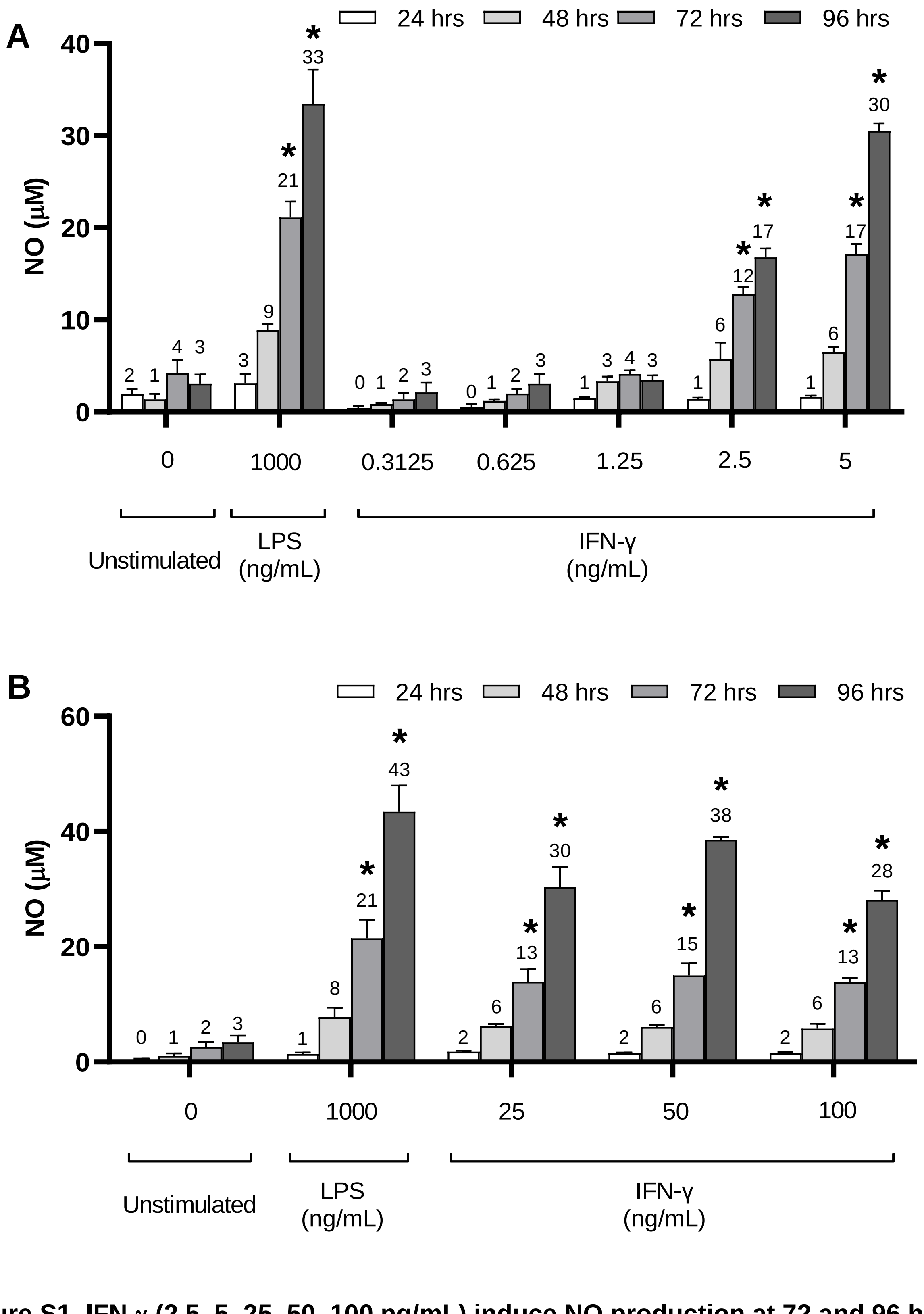

Figure S1. IFN-γ (2.5, 5, 25, 50, 100 ng/mL) induce NO production at 72 and 96 h. BV2 cells were seeded at 1.5625 x 10^4 cells/cm^2 in a 96-well plate and stimulated with LPS (1000 ng/mL), IFN-γ (0.3125, 0.625, 1.25, 2.5, 5 ng/mL and 25, 50, 100 ng/mL). The supernatant was collected and assayed for NO production at 24, 48, 72 and 96 h post-stimulation. (**A**) NO production of IFN-γ (0.3125, 0.625, 1.25, 2.5, 5 ng/mL) stimulated cells. (**B**) NO production of IFN-γ (25, 50, 100 ng/mL) stimulated cells. Results are expressed as mean ± SD of one independent experiment with at triplicates. *$p<0.0001$ compared to unstimulated cells of each group of stimulants; One-way ANOVA with Tukey's post hoc test.
