# Supplementary material for: Response Profiles of BV2 Microglia to IFN-γ and LPS Co-Stimulation and Priming
Source: Biomedicines. 2023 Sep 27;11(10):2648. doi: 10.3390/biomedicines11102648 (PMC10604055; doi:10.3390/biomedicines11102648)
Supplement: Supplementary file 1 [file biomedicines-11-02648-s001.zip › Figure S2.pdf]

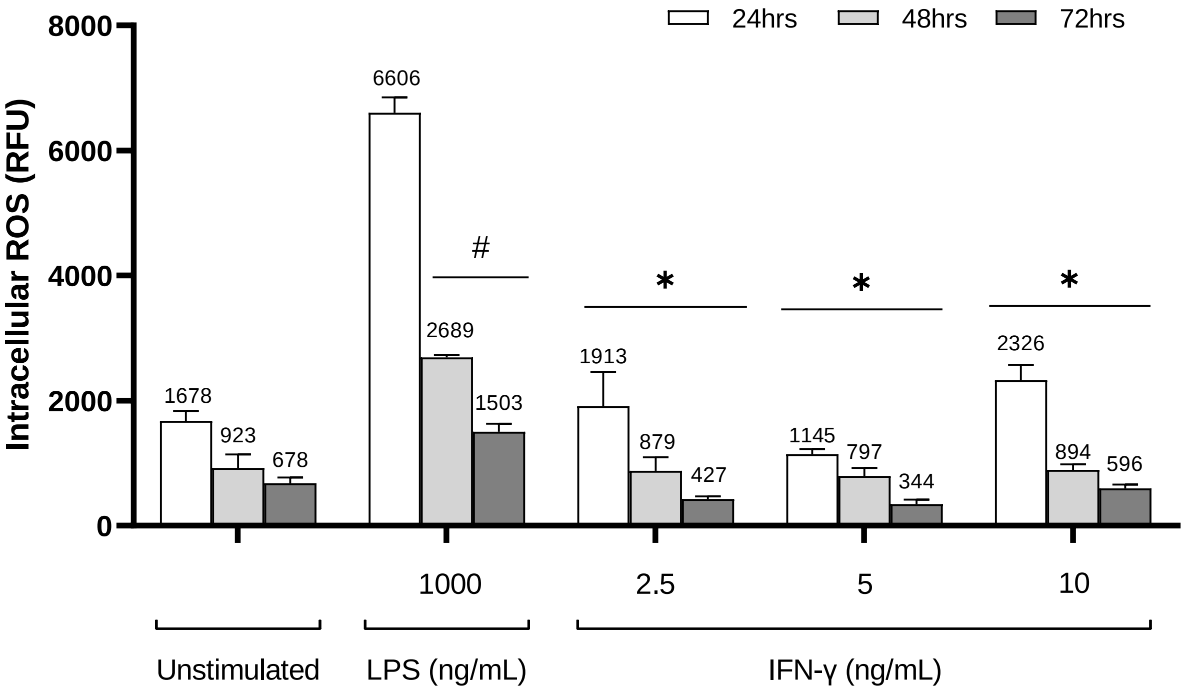

Figure S2. IFN-γ does not stimulate substantial amounts of intracellular ROS. BV2 cells at 6.25 x 10^4 cells/cm^2 were seeded in a 96-well plate and stimulated with 1000 ng/mL LPS or IFN-γ (2.5, 5 and 10 ng/mL) for 24, 48 and 72 h. Intracellular ROS was determined using the H_2DCFDA assay. Results are expressed as mean ± SD of one independent experiment with triplicates. *$p<0.0001$ compared to 1000 ng/mL LPS-stimulated cells at each respective time point. #$p<0.005$ compared to 1000 ng/mL LPS-stimulated cells at 24 h; One-way ANOVA with Tukey's post hoc test.
