# Supplementary material for: Response Profiles of BV2 Microglia to IFN-γ and LPS Co-Stimulation and Priming
Source: Biomedicines. 2023 Sep 27;11(10):2648. doi: 10.3390/biomedicines11102648 (PMC10604055; doi:10.3390/biomedicines11102648)
Supplement: Supplementary file 1 [file biomedicines-11-02648-s001.zip › Figure S3.pdf]

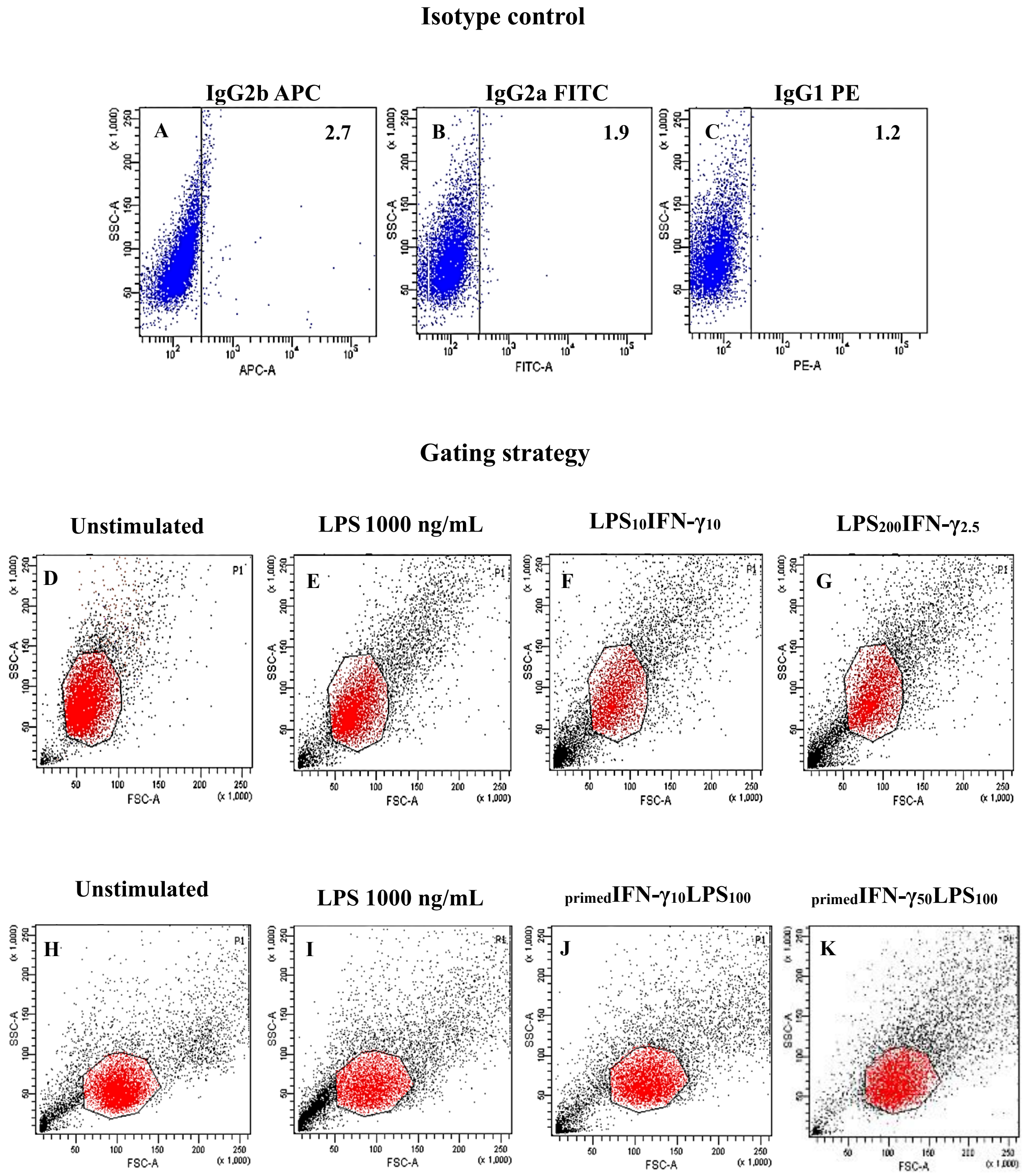

Figure S3. Isotype control and gating strategy for immunophenotyping analysis of BV2 cells. Density plots show isotype control staining for IgG2b APC (**A**), IgG2a FITC (**B**) and IgG1 PE (**C**) fluorochromes to correct for non-specific binding of antibodies. Numbers within plots indicate the percentage of positivity to each isotype control. The scatter plots show side scatter (SSC) versus forward scatter (FSC) profiles of the gated BV2 microglia cells for LPS/IFN-γ co-stimulation (**D – G**) and $_{primed}$IFN-γ/LPS priming (**H – K**).
